# Refractory *Clostridium difficile* Infection Successfully Treated with Tigecycline, Rifaximin, and Vancomycin

**DOI:** 10.1155/2012/702910

**Published:** 2012-07-09

**Authors:** Dominador Lao, Tom Chiang, Eric Gomez

**Affiliations:** ^1^St. Matthew's School of Medicine, USA; ^2^Veterans Affairs New Jersey Healthcare System (VA NJHCS), East Orange, NJ 07018, USA; ^3^Department of Infectious Disease, Appalachian Regional Hospital, Beckley, WV 25801, USA

## Abstract

The occurrence of *Clostridium difficile* colitis is on the rise and has become more difficult to manage with standard therapy. Thus, the need for alternative treatments is essential. Tigecycline is a glycylcycline antibiotic that has been shown to be effective against *C. difficile* through several published case reports and in *in vitro* studies. We present a case of *C. difficile* colitis that failed to respond to metronidazole and oral vancomycin therapy, but improved on a combination of rifaximin, tigecycline, and vancomycin.

## 1. Case Report

A 68-year-old Caucasian male with a history of multiple myeloma on lenalidomide/dexamethasone, diabetes, and end-stage renal disease on hemodialysis was admitted to the hospital for severe diarrhea and dehydration. On physical examination, he was found to be afebrile with benign abdominal exam. His white blood cell count (WBC) was normal. Fecal leukocytes were detected. Stool ova and parasites and bacterial stool cultures were negative. A computed tomography (CT) of the abdomen and pelvis showed diffuse thickening of the colon wall involving the entire colon and rectum consistent with infectious colitis. With the high suspicion of *Clostridium difficile* associated colitis, metronidazole 500 mg orally every 8 hours was empirically started. The cytomegalovirus serum polymerase chain reaction (PCR), enzyme immunoassay (EIA) for *C. difficile* toxin A/B and *C. diffile* PCR were all negative. Despite metronidazole treatment, his diarrhea persisted and on the 7th hospital day, he had a temperature of 101.8°F and rifaximin 550 mg orally twice daily was added. He responded rapidly to the combination treatment and the diarrhea and fever resolved by the 14th hospital day and he was discharged on metronidazole for 14 days. However, a week later the patient was readmitted to the hospital with diarrhea, nausea and abdominal pain. On physical examination, he was afebrile and appeared to be in mild distress with right-lower quadrant and suprapubic tenderness on palpation. His leukocyte count was 6,200 cells/*μ*L and a repeat abdominal CT showed changes of pancolitis consistent with an infectious process. Metronidazole was continued and cefepime 1 gram intravenously every 24 hours was added. Stool culture showed only normal fecal flora and blood cultures were negative, however; the EIA *C. difficile* toxin stool assay was positive. His therapy was changed from metronidazole and cefepime to vancomycin 250 mg orally every 6 hours, as his symptoms restarted within 5 days of receiving rifaximin and while on metronidazole. Over the following nine days, the patient showed little response to therapy, averaging six loose bowel movements a day along with persistent abdominal tenderness and distention. He had a WBC count of 5,000 cells/*μ*L with 32% bands, and a lactate level was elevated at 4 mmol/L. An abdominal X-ray showed no bowel distention. By the 10th hospital day, the patient developed leukocytosis with a WBC count of 17,200 cells/*μ*L and was now considered to have failed vancomycin treatment. Tigecycline 100 mg intravenously once, followed by 50 mg every 12 hours and rifaximin 550 mg orally every 12 hours, was added to the vancomycin. Though the patient's leukocyte count peaked at 19,900 cells/*μ*L (during 2nd day of modified therapy), he responded well with a gradual decrease in leukocytosis, diarrhea, and abdominal pain. He was discharged on the 16th hospital day with resolution of symptoms after 4 days of tigecycline, rifaximin, and vancomycin treatment. He was continued on this antibiotic regimen for an additional ten days. One month after finishing antibiotics, the patient was free of symptoms.

## 2. Discussion

In the past decade, there has been an increase in the incidence of *C. difficile* infections (CDI) in North America in association with the spread of 027/NAP1/BI strain [1]. This epidemic strain has been associated with more severe disease and higher rate of recurrences. Oral vancomycin and metronidazole are considered the antimicrobial of the current standard of care; however recently there have been an increased number of reports of treatment failure with these drugs, especially metronidazole [2]. In a systematic review, Vardakas et al. reported a metronidazole and vancomycin treatment failure of 22.4% and 14.2% [3]. This high rate of clinical failure with these antimicrobials and the concerns *C. difficile* strains with decreased susceptibility to metronidazole and vancomycin [1] have prompted the use on nonconventional antimicrobial agents. 

In this paper, we describe a case of vancomycin and metronidazole treatment failure that was successfully managed with the combination of rifaximin, tigecycline, and oral vancomycin. Rifaximin is an oral antibiotic which acts exclusively in the gastrointestinal tract. Though it does possess broad coverage against most Gram-positive organisms (including *C. difficile*) and nearly all enteric Gram-negative pathogens, it seems to have a little effect on normal gastrointestinal flora [4, 5] which is known to be protective for the development of CDI [6]. Rifaximin appears to be efficacious in treating CDI when used in conjunction with other antibiotics active against *C. difficile*. It has been used effectively with vancomycin via simultaneous administration, drug cycling, or sequential monotherapy [7, 8]. Also, El-Herte et al. reported a recent case of *C. difficile* in which the patient initially failed metronidazole and vancomycin therapy, but was successfully treated with a combination of rifaximin and tigecycline [9]. 

Tigecycline, a derivative of minocycline, is also a broad spectrum antibiotic active against Gram-positive and Gram-negative organisms. In addition to being indicated for the treatment of complicated skin and intra-abdominal infections, its effectiveness against *C. difficile* has been shown through several studies which reported consistently low-minimum inhibitory concentration (MIC)_90_ values ranging from 0.06 to 0.25 *μ*g/mL [10–13]. Although tigecycline was shown to decrease the number of microflora in a human gut model, there was no proliferation of *C. difficile* or increased production of its associated cytotoxin [12]. Similar results were reported in a study using a mouse model attributable to tigecycline's relative sparing of native anaerobic bacteria and its inhibitory effect on *C. difficile* [14]. 

Several reports have been published describing the treatment of *C. difficile* colitis with tigecycline. There have been 8 cases in total, 7 of which were successful in treating refractory CDI using tigecycline as monotherapy [15, 16] or in combination with another antibiotics such as metronidazole [17] or rifaximin [9]. These 7 cases reported patient recovery within one week of therapy without recurrence of symptoms after a 3 month period. On the other hand, Kopterides et al. reported clinical failure in a patient with CDI treated with tigecycline for 14 days [18]. 

In conclusion, *C. difficile *colitis patients refractory to standard treatments are on the rise. It has become more crucial to find alternative approaches for treating such cases. Although data supporting the use of tigecycline for CDI is still lacking, this paper along with several others suggest that it may be an alternative for refractory or severe infections unresponsive to standard therapy. Not only have *in vitro* studies provided evidence to support this claim, but the literature reviewed here along with the case report demonstrated successful treatment in 8 out of 9 cases using tigecycline as monotherapy or in combination with other CDI antibiotics. Nevertheless, further investigations are needed to evaluate the safety and efficacy of newer antimicrobial agents such as tigecycline for routine treatment of refractory CDI.
